# Analysis of the* UGT1A1* Genotype in Hyperbilirubinemia Patients: Differences in Allele Frequency and Distribution

**DOI:** 10.1155/2019/6272174

**Published:** 2019-07-29

**Authors:** Xiao-xiao Mi, Jian Yan, Xiao-jie Ma, Ge-li Zhu, Yi-dan Gao, Wen-jun Yang, Xiao-wen Kong, Gong-ying Chen, Jun-ping Shi, Ling Gong

**Affiliations:** ^1^Institute of Translational Medicine, The Affiliated Hospital of Hangzhou Normal University, Hangzhou, Zhejiang, China; ^2^Department of Infectious Disease (Liver Diseases), The Affiliated Hospital of Hangzhou Normal University, Hangzhou, Zhejiang, China; ^3^Department of Pathology, The Affiliated Hospital of Hangzhou Normal University, Hangzhou, Zhejiang, China

## Abstract

**Objective:**

The spectrum of* UDP-glucuronyl transferase A1 *(*UGT1A1*) variants in hereditary unconjugated hyperbilirubinemia varies markedly between different ethnic populations. This study evaluated the* UGT1A1* genotypes in hyperbilirubinemia patients from southeastern China.

**Methods:**

We enrolled 60 patients from southeastern China (44 men and 16 women; age range: 3–76 years) with unconjugated hyperbilirubinemia and performed genetic analysis of the* UGT1A1* gene by direct sequencing.

**Results:**

For patients with Gilbert syndrome, 85% (47/55) harbored pathogenic variants of* UGT1A1*⁎*60*. Both* UGT1A1*⁎*28* and* UGT1A1*⁎*81* were detected in the promoter region of* UGT1A1*. Additionally, 83% (20/24) of patients with Gilbert syndrome heterozygous for* UGT1A1*⁎*60 *had an association with heterozygous variation of* UGT1A1*⁎*28 *or* UGT1A1*⁎*81*, while 91% (21/23) of Gilbert syndrome patients homozygous for* UGT1A1*⁎*60 *had biallelic variations of* UGT1A1*⁎*28* and* UGT1A1*⁎*81*. We detected 213* UGT1A1* allelic variants, including six novel variations, with the most frequent allele being the* UGT1A1*⁎*60*, followed by* UGT1A1*⁎*28* and* UGT1A1*⁎*6*. All of the patients showed multiple sites of variants in* UGT1A1*; however, variation number was not associated with bilirubin levels (*P*>0.05).

**Conclusions:**

The spectrum of* UGT1A1 *variants in southeastern Chinese patients was distinct from other ethnic populations. Our findings broaden the knowledge concerning traits associated with* UGT1A1 *variants and help profile genotype–phenotype correlations in hyperbilirubinemia patients.

## 1. Introduction

Hereditary unconjugated hyperbilirubinemia is autosomal recessive disorder and can be categorized as Crigler–Najjar syndrome type I (CN-I; OMIM#218800), Crigler–Najjar syndrome type II (CN-II; OMIM#606785), or Gilbert syndrome (GS; OMIM#143500) based on serum bilirubin levels. The concentration of serum total bilirubin (TBIL) in CN-I, CN-II, and GS ranges from 513 *μ*M to 855 *μ*M, 102.6*μ*M to 342 *μ*M, and 17 *μ*M to 85 *μ*M, respectively [1]. These hyperbilirubinemias result from increased water-insoluble unconjugated bilirubin in the liver in the absence of liver dysfunction or hemolysis [2]. The common clinical presentation in hyperbilirubinemia patients is jaundice, and in CN-I patients, jaundice is apparent from birth and progressively accumulates to present a risk of kernicterus [3]. Under normal conditions, unconjugated bilirubin is conjugated to water-soluble bilirubin-glucuronide conjugates and secreted into bile [4].

UDP-glucuronyl transferase (UGT), encoded by* UGT1A1*, is the only enzyme in liver that glucuronidates bilirubin. Hereditary unconjugated hyperbilirubinemia, including CN-I, CN-II, and GS, is, respectively, caused by mutations in* UGT1A1 *(OMIM*∗*191740), which is a member of the UGT1 superfamily and located on chromosome (2q37). The* UGT1A1 *promoter contains a TATA-box sequence, with an open reading frame of 1062 bp length [5, 6]. UGT1A1 enzyme activity can be increased by phenobarbital administration, which induces* UGT1A1 *expression by binding to the phenobarbital-responsive module (PBREM) in the distal enhancer element [7]. To date, >130 variants in both the regulatory and coding regions of* UGT1A1* have been identified in hereditary hyperbilirubinemia patients [8], with variations identified in CN-I, CN-II, and GS reducing UGT1A1 enzyme activity to 0%, 10%, and 30%, respectively [9–11].

The spectrum of* UGT1A1 *variants varies markedly in different populations. In Caucasian populations, the most common genotype is a TA insertion in the TATA-box sequence of the* UGT1A1* gene (*UGT1A1∗*28), resulting in A(TA)7TAA instead of the normal A(TA)6TAA sequence [12, 13]. In Western countries, the allelic frequency of the TA insertion can be as high as 0.4 [14, 15], and in Asian countries, such as Japan, the most common variation is the* UGT1A1∗*6 variant in exon 1, resulting in a p.Gly71Arg substitution [16]; however, few studies have reported* UGT1A1 *variants in hyperbilirubinemia patients from China [17, 18]. Allelic differences in* UGT1A1 *in a Chinese population with hyperbilirubinemia are expected; therefore, the present study investigated the allelic frequency and distribution of* UGT1A1 *variants in southeastern Chinese patients with hyperbilirubinemia.

## 2. Methods

### 2.1. Patients

Sixty patients with unconjugated hyperbilirubinemia from southeast China were enrolled at The Affiliated Hospital of Hangzhou Normal University between 2016 and 2018. All patients showed TBIL levels ≥17.1 *μ*M, with normal liver enzymes and no evidence of hemolysis. The patients included 44 men and 16 women (age range: 3–76 years), with most originally suspected as having hyperbilirubinemia because of apparent jaundice, whereas others were admitted during conventional health checks. The patients enrolled were all checked negative for viral hepatitis, including serology tests for hepatitis A virus (HAV), hepatitis B virus (HBV), hepatitis C virus (HCV), hepatitis D virus (HDV), and hepatitis E virus (HEV). Other hepatic diseases which may cause hyperbilirubinemia were excluded, including hemolysis, alcoholic liver disease, and autoimmune liver disease. All subjects included in this study had normal levels of liver enzymes (ALT:1-52 U/L; AST:1-40 U/L). Previous/past drug history of potentially hepatotoxic medications was also excluded. Abdominal ultrasound images for all patients were normal, and no treatment was administered when the biomedical parameters were obtained. Serum TBIL levels in all 60 patients ranged from 28.8 *μ*M (1.68 mg/dL) to 301.2 *μ*M (17.61 mg/dL), with none showing TBIL levels ≥ 30 mg/dL, as seen in CN-I. Based on serum TBIL levels, 55 patients were divided into the GS group (hyperbilirubinemia: 17–85 *μ*M), three into the CN-II group (hyperbilirubinemia: 102.6–342 *μ*M), and two into the Intermediate group (borderline CN-II and GS).

Written informed consent was obtained from participants or their legal guardians. The study was approved by the Ethics Committee of the Affiliated Hospital of Hangzhou Normal University.

### 2.2. Genomic DNA Extraction and Mutation Analysis

Genomic DNA was extracted from the peripheral blood leukocytes of all patients using a genomic DNA purification kit (Qiagen, Hilden, Germany). All exon, flanking-intron, promoter, and PBREM regions of* UGT1A1 *were amplified from genomic DNA. Primers were designed using Primer Premier 5 software (http://www.premierbiosoft.com/primer design/) according to the reference cDNA sequence of* UGT1A1 *(NM_000463). Polymerase chain reaction (PCR) analysis was performed using ~100 ng genomic DNA under the following conditions: initial denaturation for 5 min at 95°C, followed by 35 cycles of denaturation at 95°C for 1 min, annealing at 58°C for 1 min, and elongation at 72°C for 1 min, with a final elongation at 72°C for 5 min. PCR products were directly sequenced on an ABI3730XL sequencer (Applied Biosystems, Foster City, CA, USA). Primers sequences used to amplify* UGT1A1 *DNA fragments were listed as Table S1.

### 2.3. Statistical Analysis

Statistical tests were performed using SPSS (v.17.0; SPSS Inc., Chicago, IL, USA). Continuous variables [age, alanine aminotransferase (ALT), aspartate aminotransferase (AST), TBIL, direct bilirubin (DBIL), and unconjugated bilirubin (IBIL)] were evaluated using the Kolmogorov–Smirnov test or the Shapiro–Wilk test for normal distribution analysis. Continuous variables that were normally distributed were expressed as the mean ± standard deviation and compared by one-way analysis of variance. Continuous variables not normally distributed were presented as the median and range and compared using the Kruskal–Wallis H test. Categorical variables were analyzed using the Chi-square test. A* P*<0.05 was considered significant.

## 3. Results

### 3.1. Patient Characteristics Based on the c.-3279T>G Genotype

Demographic information and biochemical parameters are presented in Table 1. Among the 55 GS patients, 43% (24/55) patients harbored one c.-3279T>G variation (*UGT1A1∗60*), 42% (23/55) harbored two c.-3279T>G variations, and 15% (8/55) showed no c.-3279T>G variation. Based on the c.-3279T>G genotype, we subdivided GS patients into three groups: heterozygotes with one c.-3279T>G variation, homozygotes with two c.-3279T>G variations, and wild-type (no c.-3279T>G variation harbored). Forty-one GS patients were male, including 19 heterozygotes, 17 homozygotes, 5 wild-types. There was no significant difference in gender distribution among the three subgroups of GS patients (*P*=0.54).

The age at onset in our patients with hyperbilirubinemia ranged from 3 to 76 years, and among the three subgroups of GS patients, there was no significant difference in onset age (*P*=0.25). Additionally, differences in levels of ALT (*P*=0.80), AST (*P*=0.10), albumin (*P*=0.18), and gamma-glutamyltransferase (*P*=0.09) were not significant; however, TBIL and especially IBIL levels were beyond the normal range in all GS patients, although we found no significant difference in these levels among the three subgroups. Moreover, we also detected one or two c.-3279T>G variations carried by our Intermediate patients but not CN-II patients. These findings indicated that c.-3279T>G variation is essential for the pathogenesis of mild hyperbilirubinemia.

### 3.2. Variants in the Proximal Promoter Region of* UGT1A1*

**Figure 1:**
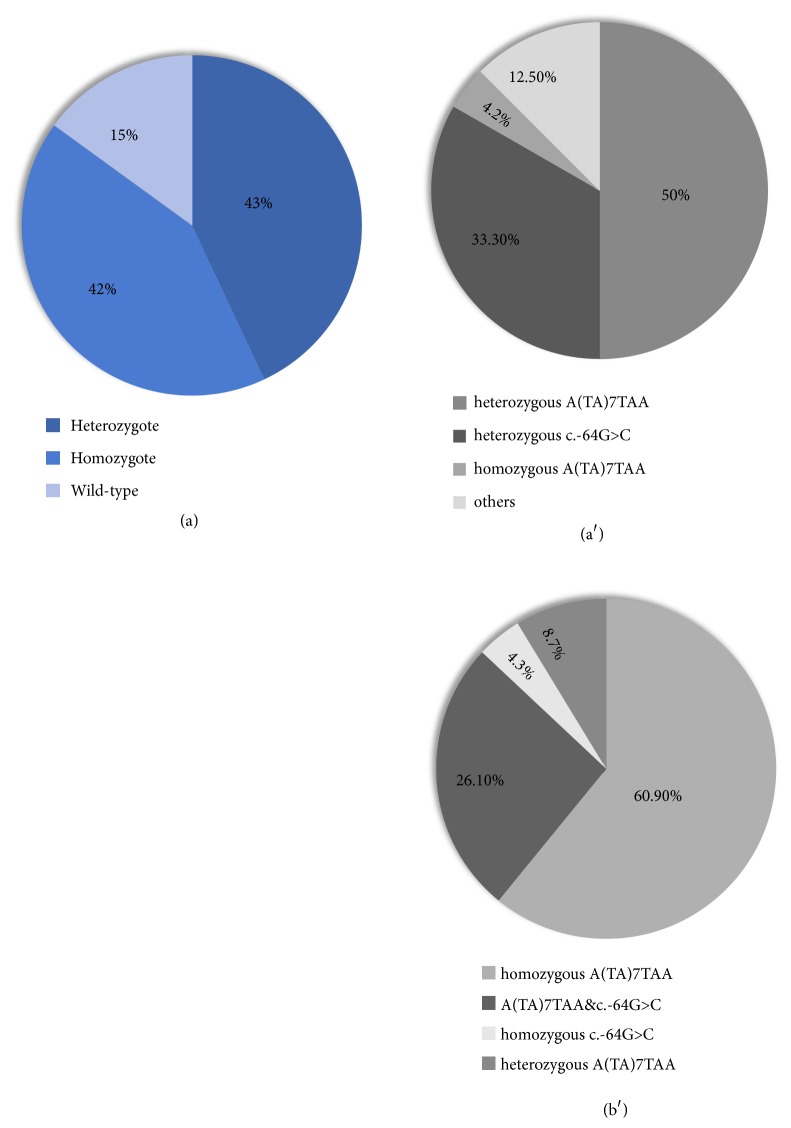
*Incidence of the c.-3279T>G genotype in GS patients*. (a) Incidence of the c.-3279T>G genotype in GS patients. (a′) Incidence of different genotypes in GS patients heterozygous for c.-3279T>G. (b′) Incidence of different genotypes in GS patients homozygous for c.-3279T>G.

As noted, 85% patients (47/55) of GS patients harbored one or two c.-3279T>G variations in the PBREM region of* UGT1A1 *(Figure 1(a)).  Table 2 shows that, of the GS patients heterozygous for the c.-3279T>G variation (*n*=24), 50% (12/24) were also heterozygous for A(TA)7TAA (*UGT1A1∗28*), 33.3% (8/24) were heterozygous for a c.-64G>C variation (*UGT1A1∗81*), one patient harbored a biallelic TA insertion, and 12.5% (8/24) showed no variations in the promoter region. These results indicated that 83.3% of GS patients heterozygous for the c.-3279T>G variation also harbored heterozygous variation in the* UGT1A1* promoter region (Figure 1(a′)), suggesting that c.-3279T>G heterozygosity is mostly accompanied by heterozygous variations in the* UGT1A1* promoter in our patient cohort.

In GS patients homozygous for the c.-3279T>G variation (*n*=23), 61% (14/23) were also homozygous for A(TA)7TAA, 4% (1/23) were homozygous for the c.-64G>C variation, 26% (6/23) harbored a TA insertion and the c.-64G>C variation, and two patients were heterozygous for the TA insertion. These results indicated that 91% of GS patients homozygous for the c.-3279T>G variation also harbored biallelic variations in the* UGT1A1 *promoter region (Figure 1(b′)), suggesting that c.-3279T>G homozygosity was frequently associated with homozygous variations in the* UGT1A1* promoter. Furthermore, in our Intermediate patients harboring the c.-3279T>G variation, we also detected a TA insertion. These findings demonstrated that the c.-3279T>G genotype was closely accompanied by A(TA)7TAA or c.-64G>C genotype in the* UGT1A1* promoter, indicating that variants of the c.-3279T>G and A(TA)7TAA or c.-64G>C represented the principal genotype associated with GS in this cohort.

### 3.3. Novel Variants

**Figure 2:**
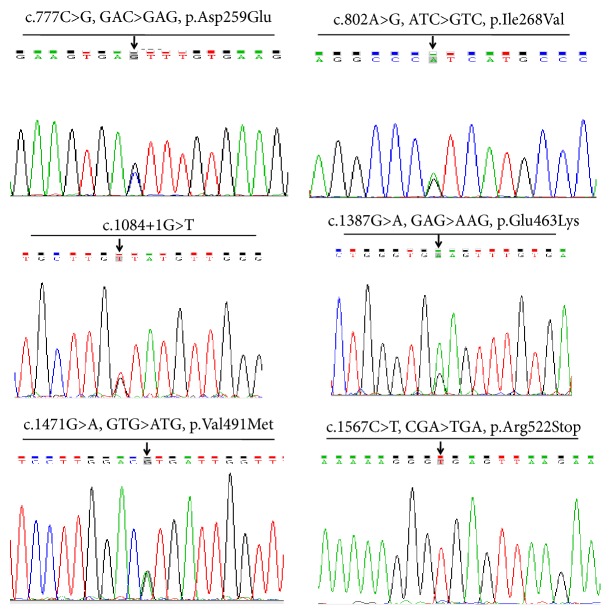
Novel variants found in 60 patients with hyperbilirubinemias.

**Figure 3:**
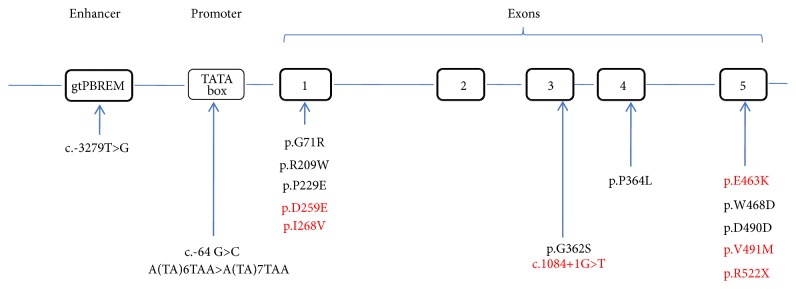
*The distribution of variants in 60 patients with hyperbilirubinemias*. Variants in* UGT1A1 *regulatory regions are shown as nucleotide changes. Variants in the* UGT1A1 *coding region are shown as amino acid substitutions. Novel variants are indicated in red.

**Figure 4:**
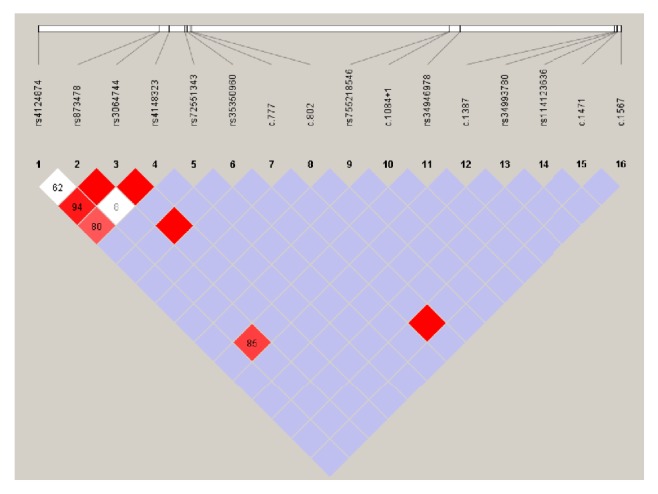
*Linkage disequilibrium analysis of the UGT1A1 variants detected in this cohort*. Pairwise LD map, a denser color indicates greater linkage.

A total of 213 allelic variants at six sites in* UGT1A1* were detected in our patient cohort, including variants in the PBREM, proximal promoter, and coding regions (exons 1, 3, 4, and 5). The most common variants were c.-3279T>G in the PBREM region, with an allele frequency of 34.3% (*UGT1A1∗60*, 73/213), followed by A(TA)7TAA in the promoter region (*UGT1A1∗28*, 52/213) and p.Gly71Arg in exon 1 (*UGT1A1∗6*, 37/213). Six novel variants were detected (Figure 2 and Table 3), including p.Asp259Glu, p.Ile268Val, c.1084+1G>T, p.Glu463Lys, p.Val491Met, and p.Arg522Stop, with all of these located in or adjacent to the coding region (Figure 3). Allelic number of these novel alleles has not been noted by UGT Nomenclature. Also linkage disequilibrium analysis was performed among all* UGT1A1* variants detected in this cohort (Figure 4).

### 3.4. Multiple Variants

**Figure 5:**
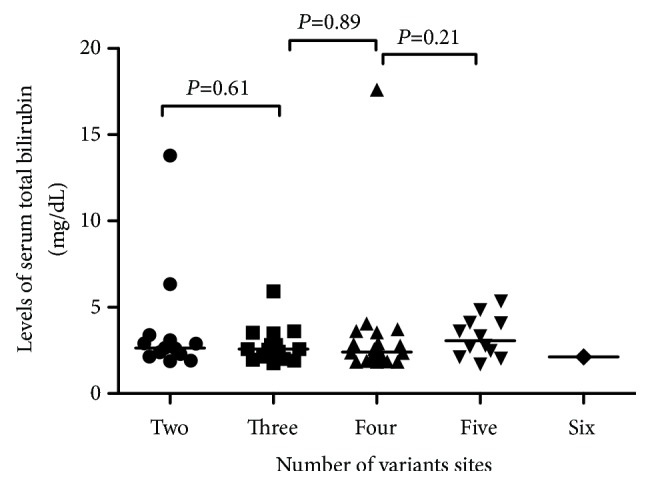
*Association between levels of serum total bilirubin and the number of variants in 60 patients with hyperbilirubinemia*. Analysis of two groups using the Mann-Whitney* U *test revealed no significant associations. Lines indicate the median of each group.

All of the patients harbored at least two sites of sequence variations associated with* UGT1A1*. Thirteen patients, including 11 GS and two CN-II patients, harbored variations at two sites (Table S2), 15 patients, including 14 GS and one Intermediate patient, harbored variations at three sites (Table S3), 19 patients, including 18 GS and one CN-II patient, harbored variations at four sites (Table S4), and 12 patients, including 11 GS and one Intermediate patient, harbored variations at five sites (Table S5). Additionally, we detected variations at six sites in one GS patient homozygous for a combination of* UGT1A1∗60*,* UGT1A1∗28*, and* UGT1A1∗27*. However, associations between levels of serum TBIL and the number of variations did not differ significantly between each group (Figure 5).

## 4. Discussion

In this study, we identified* UGT1A1 *variants in 60 patients with unconjugated hyperbilirubinemias, including 55 GS patients, three CN-II patients, and two Intermediate patients, based on their bilirubin levels. None of patients displayed bilirubin levels ≥ 30 mg/dL, suggesting the absence of CN-I. CN-I syndrome is extremely rare and can be fatal due to kernicterus [19, 20], with UGT1A1 enzyme activity in CN-I either absent or greatly attenuated [10].

GS is a mild, prolonged hyperbilirubinemia syndrome, with a prevalence ranging from 3% to 13% [21].* UGT1A1∗28* is the most common pathogenic variant found in GS patients, with an allelic frequency of 0.4 in Western populations [14] and often linked with* UGT1A1∗60* variant [22]. In the present study,* UGT1A1∗60 *was the most common variant found, with an allelic frequency of 0.34, which exceeded that in the Japanese population (allele frequency, 0.17) [23]. Additionally, we found that* UGT1A1∗28 *was the second most common variant, with an allelic frequency of 0.24. Moreover, we detected the* UGT1A1∗81 *(c.-64G>C) in the* UGT1A1* proximal promoter region, which has not been reported previously in an Asian population. In our GS patients, the* UGT1A1∗60 *was also mostly accompanied by* UGT1A1∗28* or* UGT1A1∗81*, suggesting that the genotype of* UGT1A1∗60 *accompanied with* UGT1A1∗28* or* UGT1A1∗81 *was essential for GS pathogenesis in this cohort, whereas in our CN-II patients, we did not detect this accompanying. This may be due to the limited number of patients enrolled in this group.

The missense variant of* UGT1A1∗6 *(p.Gly71Arg), resulting from a G>A substitution in exon 1 of* UGT1A1*,was the third most common pathogenic variant found in our cohort, with an allelic frequency of 0.17. This variant was identified in both GS and CN-II patients; however, a genotype heterozygous for* UGT1A1∗60*/*UGT1A1∗28 *(or* UGT1A1∗81*) was detected in most of the patients harboring* UGT1A1∗6* (18/19 patients). Five GS patients were identified as homozygous for* UGT1A1∗6*. These findings indicated that the p.Gly71Arg variant could be cause of hyperbilirubinemia in this cohort not only through its linkage with variants in the* UGT1A1* regulatory regions but also in isolation.

We identified six novel* UGT1A1*-associated variants in our hyperbilirubinemia patients, including four missense variants, one nonsense variant, and one splicing variant.* In silico* analysis using SIFT, Polyphen-2, and MutationTaster [24–26] predicted the variants of p.Asp259Glu, p.Glu463Lys, and p.Val491Met as being likely pathogenic while p.Ile268Val was predicted as benign (data not shown). Additionally, the p.Arg522Stop variant was predicted as pathogenic, resulting in a truncated UGT1A1 protein potentially causing nonsense-mediated mRNA decay [27]. Moreover, the c.1084+1G>T variation disrupts the splicing-donor site of intron 3 in* UGT1A1 *and was predicted to cause the expression of abnormal* UGT1A1* transcripts. All of these novel variants were found in the GS patients in our cohort, except for p.Arg522X, which was carried by one CN-II patient with a serum TBIL level of 301.2 *μ*M (17.6 mg/dL). These findings broaden the spectrum of* UGT1A1 *variants associated with hyperbilirubinemia syndrome.

The spectrum of variants identified in this study was distinct from that reported previously. We detected 213 allelic variants at six sites associated with* UGT1A1* in our patient cohort, with all of the patients harboring multiple variants sites. However, isolated heterozygous mutations were not detected, strongly supporting recessive inheritance of hyperbilirubinemia [2]. Furthermore, we found that the number of variants was unrelated to TBIL levels. In our CN-II and Intermediate patients, the more variant sites detected in coding regions, the more severity of hyperbilirubinemia presented, and in Gilbert patients, when we compared subgroups that harbored one coding variation site in total two sites harbored group and total five sites harbored group, we found that the more number of variations detected in promoter region, the higher levels of serum bilirubin presented (data not shown). These data suggested that allele frequency and distribution might be essential factors associated with the severity of hyperbilirubinemia. A Japanese study reported that variants located in* UGT1A *shared exons (exons 2 through 5) are present in 14.1% of GS patients (9/64) [28], whereas a Taiwanese study reported that variants located in* UGT1A1 *shared exons were absent from GS patients [29]. In the present study, we found that 29.1% of GS patients (16/55) harbored variants located in* UGT1A1 *shared exons. These results provide novel insight into population genetics associated with hyperbilirubinemia syndrome; however, further studies are required to elucidate the mechanisms associated with these variants.

In total, our study broadens the knowledge concerning traits associated with* UGT1A1* variations and helps profile genotype–phenotype correlations in hyperbilirubinemia patients. Based on the finding that most Gilbert patients harbored variants located in promoter or exon 1 and most CN-II patients harbored variants located in exons 2 through 5, our study emphasizes the value of* UGT1A1* genotypes in differential diagnosis of Gilbert and CN-II in everyday clinical practice. Also, our project addressed the genetic traits in hyperbilirubinemia patients from southeast China and will contribute to establishing genetic testing as a feasible and cost-effective tool to perform large-scale hyperbilirubinemia screening in the general population.

## Figures and Tables

**Table 1:** Demographic information and biochemical parameters in Gilbert patients.

	Total	-3279T>G	-3279T>G	-3279T>G	*P*
		Heterozygote	Homozygote	Wildtype	
N	55	24(43%)	23(42%)	8(15%)	
Sex(M/F)	41M/14F	19M/5F	17M/6F	5M/3F	0.64
Age, y	34(3~76)	33.5(3~66)	34.0(21~61)	46.0(18~76)	0.25
ALT(U/L)	26.73±11.85	27.87±12.76	26.13±12.34	25.00±7.76	0.80
AST(U/L)	24.05±12.02	27.83±16.73	20.47±4.97	23.00±4.95	0.10
ALB(g/L)	46.7(41.3~52.1)	47.3(41.3~52.1)	46.4(44.4~51.8)	45.0(42.8~49.0)	0.18
GGT(U/L)	18.93±6.96	18.42±7.24	18.0±6.26	24.12±7.06	0.09
TBil(*μ*mol/L)	43.9(28.8~82.9)	44.3(30.1~70.2)	42.4(28.8~82.9)	38.0(32.1~57.9)	0.39
DBil(*μ*mol/L)	11.84±3.33	12.17±3.40	11.93±3.21	10.64±3.62	0.53
IBil(*μ*mol/L)	30.7(21.4~70.4)	32.0(22.2~53.1)	30(21.4~70.4)	29.4(23.9~48.7)	0.32

Wild-type TT; Heterozygote TG; Homozygote GG

Variables were checked by Kolmogorov-Smirnov test or Shapiro-Wilk test for normal distribution analysis. Normally distributed data are expressed as mean±SD and compared by one-way ANOVA. Not normally distributed data were presented as median and range and were compared by Kruskal-Wallis H test. Categorical variables were analyzed using Chi-square test.

**Table 2:** Association of c.-3279T>G in PBREM with TA insertion or c.-64G>C in promoter region of *UGT1A1* in Gilbert patients.

GS (n=55)	c.-3279 T>G in PBREM		
	Wild-type	Heterozygote	Homozygote
	n=8	n=24	n=23
A(TA)7TAA			
Heter	0	12(50%)	2(8.7%)
Homo	0	1(4.2%)	14(60.9%)
c.-64G>C			
Heter	0	8(33.3%)	0
Homo	0	0	1(4.3%)
A(TA)7TA&c.-64G>C			
	0	0	6(26.1%)
Others			
	8	3(12.5%)	0

Wild-type TT; Heterozygote TG; Homozygote GG

**Table 3:** *UGT1A1 *variants found in all 60 patients with hyperbilirubinemias.

Gene Region	Nucleotide Change	Amino acid Change	rs Number in dbSNP database	No. of alleles	Allele Frequency (%)	1000g_CHB MAF (%)	P value
Enhancer							
PBREM	-3279 T>G		rs4124874	73	34.3	27.20	2.27E-06*∗*
Promoter							
	-64 G>C		rs873478	17	7.98	3.40	0.02316*∗*
TATA box	A(TA)6TAA> A(TA)7TAA		rs3064744	52	24.4	12.90	1.05E-17*∗*
Exon1							
	c.211 G>A	p.Gly71Arg	rs4148323	37	17.4	22.80	0.102251
	c.625 C>T	p.Arg209Trp	rs72551343	2	0.94	0.00	0.052645
	c.686 C>A	p.Pro229Glu	rs35350960	8	3.75	0.50	0.000572*∗*
	c.777 C>G	p.Asp259Glu	Novel	1	0.47	NA	NA
	c.802 A>G	p.Ile268Val	Novel	1	0.47	NA	NA
Exon3							
	c.1084 G>A c.1084+1 G>T	p.Gly362Ser	rs755218546 Novel	1 1	0.47 0.47	0 NA	0.171234 NA
Exon4							
	c.1091 C>T	p.Pro364Leu	rs34946978	9	4.22	2.40	0.018437*∗*
							
Exon5							
	c.1387 G>A	p.Glu463Lys	Novel	1	0.47	NA	NA
	c.1456 T>G	p.Tyr486Asp	rs34993780	6	2.82	0	0.000735*∗*
	c.1470 C>T	p.Asp490Asp	rs114123636	1	0.47	0.50	0.652817
	c.1471 G>A	p.Val491Met	Novel	1	0.47	NA	NA
	c.1567 C>T	p.Arg522Stop	Novel	2	0.94	NA	NA

dbSNP: database of Single Nucleotide Polymorphism(https://www.ncbi.nlm.nih.gov/SNP/);

1000g_CHB MAF: Minor allele frequency of Han population in Beijing, China in 1000 genomes database(http://www.1000genomes.org).

## Data Availability

All data generated or analysed during this study are included in this published article [and its supplementary information files].

## Abbreviations

UGT1A1: UDP-glucuronyl transferase A1
GS: Gilbert syndrome
CN-I: Crigler–Najjar syndrome type I
CN-II: Crigler–Najjar syndrome type II
PBREM: Phenobarbital-responsive module
TBIL: Total bilirubin levels
DBIL: Direct bilirubin levels
IBIL: Indirect bilirubin levels
ALT: Alanine aminotransferase
AST: Aspartate aminotransferase
ALB: Albumin
GGT: Gamma-Glutamyltransferase.

## Supplementary Materials

Supplementary Materials Table S1: primer sequences used for amplicons of the UGT1A1 gene. Table S2: patients with variations at two sites. Table S3: patients with variations at three sites. Table S4: patients with variations at four sites. Table S5: patients with variations at five sites.
