# Supplementary material for: Analysis of the UGT1A1 Genotype in Hyperbilirubinemia Patients: Differences in Allele Frequency and Distribution
Source: Biomed Res Int. 2019 Jul 29;2019:6272174. doi: 10.1155/2019/6272174 (PMC6699345; doi:10.1155/2019/6272174)
Supplement: Supplementary Materials — Table S1: primer sequences used for amplicons of the UGT1A1 gene. Table S2: patients with variations at two sites. Table S3: patients with variations at three sites. Table S4: patients with variations at four sites. Table S5: patients with variations at five sites. [file 6272174.f1.docx]

| Table S1 Primer sequences used for amplicons of the *UGT1A1* gene | | | |
| --- | --- | --- | --- |
| Amplicon | Size of PCR | Primer sequences |  |
|  | product, bp | (forward, reverse) |  |
| PBREM | 400 | 5′-CACCTCCTCCTTATTCTCTT-3′ |  |
|  |  | 5′-CTCATTCCTCCTCTCTAGCC-3′ |  |
| Promoter | 315 | 5′-GCCAGTTCAACTGTTGTTGCC-3′ |  |
|  |  | 5′-CCACTGGGATCAACAGTATCT-3′ |  |
| Exon1-1 | 603 | 5′-GGTGTATCGATTGGTTTTTGC-3′ |  |
|  |  | 5′-GGCAGTGCATGCAAGAAGA-3′ |  |
| Exon1-2 | 657 | 5′-TTGTCTGGCTGTTCCCACTT-3′ |  |
|  |  | 5′-TGCCAAAGACAGACTCAAACC-3′ |  |
|  |  | 5′-AACACGCATGCCTTTAATCATA-3′ |  |
| Exon2 | 416 | 5′-TGACAACAACCACAACAACAAA-3′ |  |
|  |  | 5′-GAAGTTGCCAGTCCTCAGAA-3′ |  |
| Exon3 | 459 | 5′-TGTTGGCCATAATATTTTCAAGC-3′ |  |
|  |  | 5′-AACACTGAGTCTTTGGAGTGTTTTC-3′ |  |
| Exon4 | 420 | 5′-TATTTGAAACAACGCTATTAAATGCT-3′ |  |
|  |  | 5′-CAGGTTTCCTTTCCCAAGTTT-3′ |  |
| Exon5 | 625 | 5′-GGGGGCACGATACATATTCA-3′ |  |

Table S2 Patients with variations at two sites

| *UGT1A1* variants |  | GS | Intermediate | CN-II | Total (n) |
| --- | --- | --- | --- | --- | --- |
| Two variant sites |  |  |  |  |  |
| -3279 T>G |  |  |  |  |  |
|  | A(TA)7TAA | 1 | 0 | 0 | 1 |
|  | p.G71R | 1 | 0 | 0 | 1 |
|  | p.P364L | 1 | 0 | 0 | 1 |
|  | p.E463K | 1 | 0 | 0 | 1 |
| p.G71R |  |  |  |  |  |
|  | -64 G>C | 1 | 0 | 0 | 1 |
|  | p.G71R | 5 | 0 | 0 | 5 |
|  | p.Y486D | 1 | 0 | 0 | 1 |
| p.R209W |  |  |  |  |  |
|  | p.R209W | 0 | 0 | 1 | 1 |
| p.Y486D |  |  |  |  |  |
|  | p.Y486D | 0 | 0 | 1 | 1 |
| Total (n) |  | 11 | 0 | 2 | 13 |

Table S3 Patients with variations at three sites

| *UGT1A1* variants |  | GS | Intermediate | CN-II | Total (n) |
| --- | --- | --- | --- | --- | --- |
| Three variant sites |  |  |  |  |  |
| -3279 T>G+  A(TA)7TAA |  |  |  |  |  |
|  | -3279 T>G | 1 | 0 | 0 | 1 |
|  | A(TA)7TAA | 1 | 0 | 0 | 1 |
|  | p.G71R | 6 | 0 | 0 | 6 |
|  | p.P229E | 1 | 0 | 0 | 1 |
|  | c.1084+1 G>T | 1 | 0 | 0 | 1 |
| -3279 T>G+  c.-64 G>C |  |  |  |  |  |
|  | p.G71R | 2 | 0 | 0 | 2 |
|  | p.Y486D | 1 | 0 | 0 | 1 |
| -3279 T>G+  p.Y486D |  |  |  |  |  |
|  | p.G362S | 0 | 1 | 0 | 1 |
| p.G71R |  |  |  |  |  |
|  | p.I268V | 1 | 0 | 0 | 1 |
| Total (n) |  | 14 | 1 | 0 | 15 |

Table S4 Patients with variations at four sites

| *UGT1A1* variants |  | | GS | Intermediate | CN-II | Total (n) |
| --- | --- | --- | --- | --- | --- | --- |
| Four variant sites | |  |  |  |  |  |
| -3279 T>G+  A(TA)7TAA | |  | 9 | 0 | 0 | 9 |
| -3279 T>G+  A(TA)7TAA+  -64 G>C | |  | 1 | 0 | 0 | 1 |
| -3279 T>G+  A(TA)7TAA+  p.G71R | |  |  |  |  |  |
|  | | p.Y486D | 1 | 0 | 0 | 1 |
|  | | p.D490D | 1 | 0 | 0 | 1 |
| -3279 T>G+  c.-64 G>C+  p.G71R | |  |  |  |  |  |
|  | | -3279T>G | 1 | 0 | 0 | 1 |
|  | | p.G71R | 2 | 0 | 0 | 2 |
|  | | p.P364L | 3 | 0 | 0 | 3 |
| p.G71R | |  |  |  |  |  |
|  | | p.R522X | 0 | 0 | 1 | 1 |
| Total (n) | |  | 18 | 0 | 1 | 19 |
|  | |  |  |  |  |  |

Table S5 Patients with variations at five sites

| *UGT1A1* variants |  | GS | Intermediate | CN-II | Total (n) |
| --- | --- | --- | --- | --- | --- |
| Five variant sites |  |  |  |  |  |
| -3279 T>G+  A(TA)7TAA |  |  |  |  |  |
|  | p.P229Q | 4 | 1 | 0 | 5 |
| -3279 T>G+  c.-64 G>C |  |  |  |  |  |
|  | p.P364L | 1 | 0 | 0 | 1 |
| -3279 T>G+  A(TA)7TAA+  -64 G>C |  |  |  |  |  |
|  | p.G71R | 1 | 0 | 0 | 1 |
|  | p.P364L | 4 | 0 | 0 | 4 |
| -3279 T>G+  A(TA)7TAA+  p.G71R |  |  |  |  |  |
|  | p.D259E  p.V491M | 1 | 0 | 0 | 1 |
| Total (n) |  | 11 | 1 | 0 | 12 |
